# Quillaia Toothwash

**Published:** 1881-02

**Authors:** Alexander E. Bennett


### QUILLAIA TOOTHWASH.

BY ALEXANDER E. BENNETT, PH. G.

An excellent toothwash containing glycerin is made as follows: R. Soap bark, ground, 4 ounces; glycerin, 3 ounces; diluted alcohol, sufficient for 2 pints; oil of gaultheria, oil of peppermint, *a a.* 20 drops.

Macerate the soap bark in the mixture of glycerin and diluted alcohol for three or four days, and filter through a little magnesia previously triturated with the volatile oils.

Thus made, a much better preparation is obtained than by macerating the bark in the dilute alcohol, and adding the glycerin afterward.—*American Journal of Pharmacy.*

